# CD4 Lymphocyte Enumeration and Hemoglobin Assessment Aid for Priority Decisions: A Multisite Evaluation of the BD FACSPresto^™^ System

**DOI:** 10.2174/1874613601711010076

**Published:** 2017-10-24

**Authors:** Madhuri Thakar, Francis Angira, Kovit Pattanapanyasat, Alan H.B. Wu, Maurice O’Gorman, Hui Zeng, Chenxue Qu, Bharati Mahajan, Kasama Sukapirom, Danying Chen, Yu Hao, Yan Gong, Monika De Arruda Indig, Sharon Graminske, Diana Orta, Nicole d’Empaire, Beverly Lu, Imelda Omana-Zapata, Clement Zeh

**Affiliations:** 1 National AIDS Research Institute, Pune, India; 2 Kenya Medical Research Institute/US CDC Research and Public Health Collaboration, Kisumu, Kenya; 3 US Centers for Disease Control and Prevention (CDC-Kenya), Kisumu, Kenya; 4 Faculty of Medicine, Siriraj Hospital, Mahidol University, Bangkok, Thailand; 5 San Francisco General Hospital, San Francisco, CA, USA; 6 Children’s Hospital Los Angeles, Los Angeles, and The Keck School of Medicine, University of Southern California, CA, USA; 7 Beijing Ditan Hospital, Capital Medical University, Beijing, China; 8 Peking University First Hospital, Beijing, China; 9 BloodCenter of Wisconsin, Milwaukee, Wisconsin, USA; 10 BioCollection Worldwide Incorporated, Miami, Florida, USA; 11 BD Biosciences, San Jose, California, USA

**Keywords:** CD4, HIV/AIDS, Hemoglobin, Venous, Capillary, HIV-1 diversity, Recent infections, Precision

## Abstract

**Background:**

The BD FACSPresto^™^ system uses capillary and venous blood to measure CD4 absolute counts (CD4), %CD4 in lymphocytes, and hemoglobin (Hb) in approximately 25 minutes. CD4 cell count is used with portable CD4 counters in resource-limited settings to manage HIV/AIDS patients. A method comparison was performed using capillary and venous samples from seven clinical laboratories in five countries. The BD FACSPresto system was assessed for variability between laboratory, instrument/operators, cartridge lots and within-run at four sites.

**Methods:**

Samples were collected under approved voluntary consent. EDTA-anticoagulated venous samples were tested for CD4 and %CD4 T cells using the gold-standard BD FACSCalibur^™^ system, and for Hb, using the Sysmex^®^ KX-21N^™^ analyzer. Venous and capillary samples were tested on the BD FACSPresto system. Matched data was analyzed for bias (Deming linear regression and Bland-Altman methods), and for concordance around the clinical decision point. The coefficient of variation was estimated per site, instrument/operator, cartridge-lot and between-runs.

**Results:**

For method comparison, 93% of the 720 samples were from HIV-positive and 7% from HIV-negative or normal subjects. CD4 and %CD4 T cells venous and capillary results gave slopes within 0.96–1.05 and R^2^ ≥0.96; Hb slopes were ≥1.00 and R^2^ ≥0.89. Variability across sites/operators gave %CV <5.8% for CD4 counts, <1.9% for %CD4 and <3.2% for Hb. The total %CV was <7.7% across instrument/cartridge lot.

**Conclusion:**

The BD FACSPresto system provides accurate, reliable, precise CD4/%CD4/Hb results compared to gold-standard methods, irrespective of venous or capillary blood sampling. The data showed good agreement between the BD FACSPresto, BD FACSCalibur and Sysmex systems.

## 1 INTRODUCTION

Significant progress has been achieved in increasing the number of patients with HIV/AIDS receiving antiretroviral treatment (ART), reducing morbidity and mortality in these people, preventing mother-to-child transmission and extending care to remote areas [1]. Sub-Saharan Africa is home to 10% of the world’s population, but more than 60% of the world’s HIV-infected people reside there. Thus HIV/AIDS continues to pose substantial public health challenges [1, 2].

Determination of the CD4 absolute cell count (CD4) is widely recognized as a robust surrogate marker of the immune competence status in adolescent and adult HIV/AIDS cohorts [3, 4]. However, in early childhood, during development of the immune system, CD4 counts [5], therefore, the percentage of CD4 cells (%CD4) in the lymphocyte population is considered a reliable surrogate marker for children under 5 years of age [6, 7]. Point-of-care (POC) CD4 cell counters placed closer to vulnerable populations facilitate expeditious CD4+ cell testing using venous and capillary blood to enumerate CD4 cells, enabling diagnosis and initiation of treatment during a single clinic visit [8] Furthermore, monitoring and management of HIV/AIDS patients can be achieved with greater efficiency closer to the patient’s residence [8, 9].

Anemia has been identified as an additional parameter to assess the HIV-disease progression [10] and can be diagnosed by measuring the concentration of hemoglobin (Hb) in venous or capillary blood using a POC system [11]. Drug-induced anemia has been associated with exposure to antiretroviral zidovudine (ZDV) [12-14], or can also be secondary to nutrient deficiencies or concomitant conditions [15]. Close monitoring of pregnant women with pre-existing anemia and advanced HIV/AIDS is recommended [13]. Therefore a system that is easy to use and provides results in a short time would be a suitable alternative for improving HIV/AIDS early detection and treatment in remote and limited-resource facilities.

The BD FACSPresto^™^ system is a portable CD4 counter with unit-dose disposable cartridges using a single drop of venous or capillary blood for sample preparation, reporting CD4, %CD4 T cells and Hb results. The BD FACSPresto^™^ cartridge kit contains the BD FACSPresto^™^ CD4/Hb cartridge, BD Microtainer^®^ Contact-Activated Lancet, sterile alcohol prep pads, plastic adhesive bandage, sterile non-woven sponge and transfer pipets. The cartridges contain dried fluorochrome-conjugated antibody reagents (CD4 PE-Cy^™^5/ CD3 APC/CD45RA APC/CD14 PE) with integrated reagent quality control (QC), which are stable at room temperature. The BD FACSPresto instrument, with embedded software and integrated instrument quality control, uses fluorescence microscopy and absorbance spectroscopy to examine the cartridge. The turn-around time for reporting results was within 25 minutes from blood application onto the cartridge.

It is hypothesized that results for CD4, %CD4 T cells and Hb on the BD FACSPresto system are accurate and reproducible when samples are measured within 24 hours of phlebotomy. The study objectives were to demonstrate that the performance of the BD FACSPresto system is comparable to standard-of-care commercially available systems used for enumeration of CD4 cells and Hb using matched venous and capillary blood specimens from both HIV-positive and HIV-negative subjects; and to show that the results from venous and capillary blood tested on the BD FACSPresto are comparable. Specimens were prospectively collected from representative HIV-positive and HIV-negative cohorts under the care of healthcare institutions.

## 2 MATERIALS AND METHODS

**Fig. (1):**
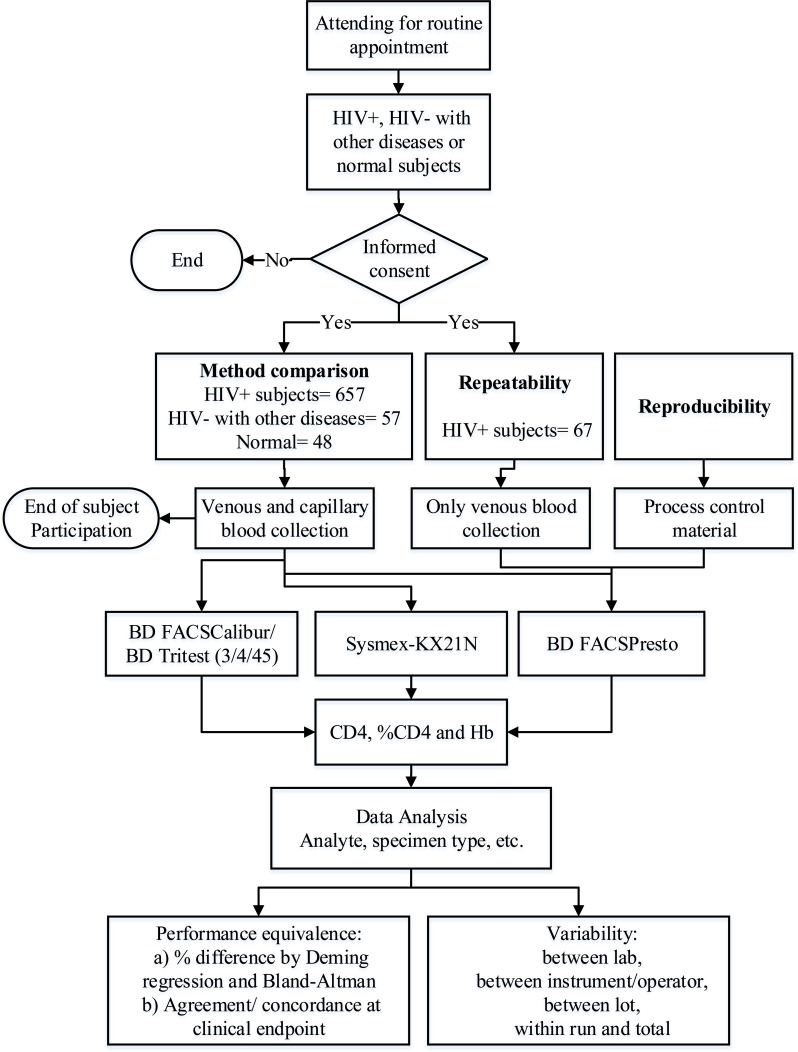
Clinical evaluation of the BD FACSPresto^™^ system flowchart. Evaluation of the performance of the BD FACSPresto system using venous and capillary blood specimens from subjects attending a routine clinic visit.

The Clinical Laboratory Standards Institute (CLSI) guidelines’ EP09-A3 method comparison, EP24-A2 bias estimation, and EP05-A3 evaluation of precision [16-18] provided the basis for the study design (Fig. **1**).

### 2.1 Ethics Review

Each site‘s Ethics Review Committee/Institutional Review Board reviewed and provided written approvals of the protocol, informed consent and minor assent (if applicable) The protocols were conducted under Good Clinical Practices and Good Laboratory Practices guidelines [19] to ensure participant safety, privacy and quality of results.

### 2.2 Specimen Acquisition

The multisite method comparison specimens were prospectively acquired from normal subjects, from HIV-positive subjects, or from HIV-negative subjects with other diseases following the study-site procedures for voluntary informed consent and minor assent. The study was carried out at seven study sites in five countries. Six sites enrolled HIV-positive patients: the HIV laboratory of the collaboration between the Kenya Medical Research Institute and the US Centers for Disease Control, Kisumu, Kenya (KEMRI/CDC); the National AIDS Research Institute (NARI), Pune, India; the Siriraj Hospital (SIR), Bangkok, Thailand; the San Francisco General Hospital (SFGH), San Francisco, California, USA; the Children’s Hospital Los Angeles (CHLA), California, USA; the Ditan Hospital (DIT), Beijing, China. The Peking Hospital (PEK), Beijing, China enrolled only HIV-negative subjects. The procedures for venous and capillary blood draw represent minimal risk to the participating subjects. The privacy of the subjects was maintained by de-linking the samples from all protected health information. The study was registered in ClinicalTrials.gov (NCT02396355; https://www.clinicaltrials.gov/ct2/show/NCT02396355?term=facspresto&rank=1). The reproducibility study was performed at SFGH, the BloodCenter of Wisconsin (BCW), Milwaukee, WI, USA, and the BD Biosciences Medical Laboratory (MED), San Jose, California, USA. The repeatability study was conducted at BioCollections Incorporated (BWI), Miami, Florida, USA.

Capillary blood specimens were collected in subjects 8 years old and older with the blue BD Microtainer (1.5-mm width x 2.0-mm depth), or for children under 8 years of age with the pink BD Microtainer (1.8 mm x 21 G) following the guideline [20]. A drop of capillary blood was immediately transferred onto the cartridge. Venous blood was collected in BD Vacutainer^®^ K2/K3 tubes with EDTA anticoagulant and transferred to the cartridge using a transfer pipet, following the manufacturer’s instructions. Subjects were observed to ensure that the bleeding through the site of skin puncture had ceased, and then were dismissed, concluding their participation in the study. Seven hundred sixty-two (762) specimens were enrolled in the method comparison study, and 67 in the repeatability study.

### 2.3 Specimen Testing

The method comparison evaluated the system’s equivalency of performance between the BD FACSPresto system and standard-of-care or predicate methods, using the same protocol: BD FACSCalibur for CD4 counts and Sysmex for Hb concentration. Clinical laboratories participated in the external quality assurance program for CD4 counts for the respective methodologies. The CD4 predicate BD FACSCalibur method was composed of the BD FACSCalibur^™^ flow cytometer, BD Tritest^™^ CD3/CD4/CD45 reagent with BD Trucount^™^ tubes and BD Multiset^™^ software. The Hb Sysmex^®^ method was the Sysmex^®^ Automated Hematology Analyzer KX-21N. Nine BD FACSPresto instruments, seven BD FACSCalibur flow cytometers, four Sysmex analyzers, and eleven lots of cartridges were used during testing. The method comparison enrollment was from January, 2014 to April, 2015.

Venous blood was further tested within 6 hours of collection using BD FACSCalibur and Sysmex instruments following the manufacturer’s instructions. Venous and capillary blood samples were tested on the BD FACSPresto system. All cartridges with samples were incubated between 18 minutes and 2 hours.

On each day of testing, the BD FACSPresto instrument was turned on, the instrument QC test automatically run, and results printed. CD4 and Hb external quality controls were run on the corresponding instruments before testing patient samples. CD4 quality controls for the BD FACSCalibur system were BD^™^ Multi-Check control and BD^™^ Multi-Check CD4 low control, and CD Chex Plus^®^ BC and CD Chex Plus^®^ BC CD4-L for the BD FACSPresto system. Three levels of Hb controls (low, normal, and high levels) were used. Sysmex EIGHTCHECK-3WP X-TRA^™^ hematology controls were used on the Sysmex system and Eurotrol^®^ 301 controls were used on the BD FACSPresto system.

The CD4 estimation using the BD FASCalibur system was carried out using a standard methodology as previously described [21, 22]. Briefly, 50 µL of venous blood was dispensed into a BD Trucount tube, 200 µL of BD Tritest CD3/CD4/CD45 reagent was added, mixed and incubated for 15 min, followed by addition of 450 µL of lysing solution and incubation for another 15 min. The stained samples were acquired in a BD FACSCalibur system using the CD3/CD4/CD45 application of the BD Multiset software. A minimum of 15,000 lymph events were collected and the CD3 and CD4 gates were revised following the manufacturer’s instructions. Hemoglobin was measured directly from the venous blood primary tube.

Only the BD FACSPresto system was used to evaluate variability during reproducibility and repeatability testing, and the control procedures described were followed on a daily basis. Enrolled HIV^+^ specimens ensured statistical representation across the assay ranges for each parameter.

The site-to-site reproducibility study was performed between September and October, 2015 at three sites using the same lot of process controls tested during five non-consecutive days, two runs per day. Repeatability testing was performed at one clinical site to evaluate variability across three operators/instruments and three cartridge lots using venous blood; one operator was assigned to the same instrument during the study. The enrolled specimens were distributed in four CD4 cell count (cells/µL) bins: low counts (≤200 cells/µL), medium counts (>200 to ≤500 cells/µL), high counts (>500 to ≤1,000 cells/µL), and very high counts (>1,000 to <5,000 cells/µL). Each specimen was prepared in each cartridge lot in duplicate and incubated. The cartridges were acquired on the three instruments.

Specimens with valid results were analyzed. Results were considered invalid if testing did not comply with the protocol procedures (inclusion or exclusion criteria, testing outside the recommended time window, *etc.*) or if system errors suppressed results.

### 2.4 Statistical Analysis

Analysis methods were in accordance with CLSI guidelines [16-18]. Data collected on case report forms and instrument files was integrated in the database. Only evaluable samples were analyzed using statistical software packages: SAS^®^ v9.3; Analyse-it^®^ v2.22; Microsoft^®^ Excel^®^ v12.0; and CBstat5 v5.1.0. Identified outliers were investigated [16] and no outliers were removed from analysis.

Venous and capillary results from BD FACSPresto instruments were analyzed independently against the results from the BD FACSCalibur or Sysmex systems. The agreement between the BD FACSPresto and predicate results was assessed by Deming regression [23] and by Bland-Altman analysis [24]. Weighted Deming regression was performed on CD4 only to stabilize the variance in the regression and to obtain an accurate estimate of the intercept. The weight for this analysis was the inverse average squared [25], because the variance increases as the counts increase over a large range. The ordinary or un-weighted Deming regression was performed on %CD4 and Hb, because the values cover a much smaller range and can be expected to have relatively similar variance. Predicted bias intervals from Deming regression were also reported around the cutoff for CD4 of 200 cells/µL [26] agreement or concordance analysis was performed around the cutoff, and the exact confidence intervals (CIs) were reported [27]. A small number of samples were excluded from analysis due to system errors.

The multisite reproducibility and repeatability data was analyzed based on the EP05-03 [18] guideline. The variance component method calculated the variance between laboratory, between instrument/operator, between lot and within run. The total variation was the sum of these components, excluding the donor variability.

## 3 RESULTS

The method comparison study results presented here are from 762 enrolled subjects, grouped in HIV+ (N = 657), HIV– with other non-HIV related diseases (N = 57), or healthy (N = 48). By specimen type, 730 were venous blood and 690 were capillary blood, with a 1.27 male: female ratio. The children (2–11 years of age) and adolescent (12–21 years of age) groups were based on the FDA guideline [28]; 7.9% were from children, 9.5% were from adolescents (12–21), and 82.6% were from subjects 22 years and older. The mean, median, and minimum and maximum values (Min-Max) were summarized by specimen type, parameter and by age group in (Table **1**).

Pooled data from all sites was used for analysis. Venous and capillary results were independently examined to show system equivalency, agreement at the CD4 cells clinical cutoff of 200 cells/µL, and system errors.

For the repeatability, additional 67 HIV+ adults between 21 and 64 years of age were enrolled in the study. The age mean was 49.5 years of age. There were 30 females and 37 males distributed in the CD4 bins: low (N=12); medium (N= 17); high (N= 25) and very high (N= 13).

### 3.1 System Equivalency

Specimens included in analysis for CD4 counts, %CD4, and Hb were grouped in predefined bins based on the analytical range of the assay. All capillary blood samples had a corresponding venous sample from the same subject. The percent bias (%bias) was calculated between the BD FACSPresto and the predicate BD FACSCalibur or Sysmex systems. Bland-Altman plots Fig. (**2**) for CD4, %CD4, and Hb mean %bias with limits of agreement for venous blood (2A, 2C, and 2E) and for capillary blood (2B, 2D, and 2F) are presented. The mean %biases for venous and capillary blood cells showed values for CD4 cells between 3.28% and 3.41%, for %CD4 between 0.2% and 2.97%, and for Hb between -2.17% and -0.47%.

The Deming regression results on CD4, %CD4 T cells, and Hb gave slope values between 0.94 and 1.05 and R^2^ ≥0.97 for CD4, between 0.99 and 1.03 and R^2^ ≥0.96 for %CD4 and, slope values were between 0.99 and 1.06 and R^2^ ≥0.89 for Hb. The Deming regression plots per specimen type and parameter are shown in (Fig. **3**).

The percent bias with limits of agreement per parameter and the Deming regression R^2^, slope and intercept are presented in (Table **2**).

The regression analysis completed in paired venous and capillary samples from the same subject gave slope values between 0.97 and 1.09 for CD4, %CD4, and Hb (Table **3**).

### 3.2 Agreement

The method agreement or concordance analysis was performed at a CD4 of 200-cells/µL cutoff in venous and capillary blood and gave an overall agreement of ≥97.7% (Table **4**).

### 3.3 Multisite Variability

Reproducibility of results are presented per site and parameter level as the mean, the percent of the coefficient of variation (%CV) with %CV upper limit (UL), or the standard deviation (SD) with SD (UL) of total precision for each control sample tested. The total precision %CV values less than 6% were consistent for CD4, less than 2.0% for %CD4, and less than 3.2% for Hb across sites (Table **5**).

Repeatability testing evaluated only venous blood (67), the variability results for between instrument/operator, between cartridge lots, within-run and total variability were grouped per CD4 bin and also per parameter in Table (**6**). In summary, the CD4 total variation per bin gave %CV values <8%. Per parameter, the total variation for CD4 gave %CV values less than 3.27%; for %CD4 and Hb the %CV values were 2.8% and 5.11%, respectively (Table **6**).

### 3.4 System Errors

If the BD FACSPresto system showed an error code during testing, the site operators were instructed to re-run the cartridge once, which often resolved the error. A cartridge failure was recorded if the second acquisition of the same cartridge did not produced valid result(s), and/or a new cartridge was used to prepare the sample.

Of the 5,442 cartridge test runs reported here, 114 cartridge runs had results suppressed and/or error codes, 99 from patient samples and 15 from process controls. Of these 114, 26 cartridges were successfully re-run. For the 69 cartridge failures, the sample was retested using a new cartridges, providing a failure rate of 1.3%. Re-running the cartridge takes an additional 3-5 min to obtain results.

## 4 DISCUSSION

The CD4-T cell absolute counts has been a broadly accepted surrogate marker [3, 9, 29] for staging and monitoring HIV+ /AIDS patients, and was used for the decision to initiate ART in adolescent and adult patients. For children under 5 years of age, %CD4 results had been considered a more reliable surrogate marker due to normal lymphocytosis during development [6]. Recently, there has been a shift in recommendation of when to measure CD4-T cell counts and how frequently since evidence strongly suggest initiation of ART is independent of the CD4 counts [30-32]. For subjects in resource-limited settings, the available CD4 POC devices have been highly useful for rapid CD4 monitoring [33-35], with some limitations for capillary blood, because the method is highly sensitive to the training and experience of the person collecting the blood [35]. The BD FACSPresto system provides results for both CD4 and %CD4 that are frequently used for monitoring co-infections, opportunistic infections, or treatment failure, and for HIV-infected populations that have no access to viral load testing [31, 32]. Obtaining the baseline CD4 cell count at the start of the ART continues to offer valuable insight of the immune system status for monitoring and long term care management, since early initiation of ART has shown to reduce the hazard ratio for serious AIDS related event [30].

Anemia is a commonly encountered hematological abnormality in HIV/AIDS with a significant impact on clinical outcomes [10, 12]. In addition, anemia is an important prognostic factor in HIV infection. ZDV is known for hematological adverse effects that include macrocytic anemia or neutropenia [14]. Determination of the concentration of Hb during ART can be an aid for early detection and monitoring of anemia in patients receiving ZDV [36] and in HIV-infected pregnant women and children [37]. Several studies have shown that Hb concentration reflects the rapidity of disease progression rates and independently predicts prognosis across demographically diverse populations [10, 12]. The rate at which Hb decreases also correlates with falling CD4 cell counts [36] suggesting that increases in Hb are predictive of ART success [36, 37]. The BD FACSPresto system includes measurement of Hb from the same sample as a third analytic parameter. The incorporation of these three parameters in the BD FACSPresto system was envisioned for more integrated and comprehensive routine monitoring of HIV/AIDS patients in resource-limited settings using capillary or venous blood.

Investigators have demonstrated equivalency of the white cell components between venous and capillary blood [38]. Recently, it was reported using a POC instrument that CD4 results were comparable to the predicate when the cartridges were filled using a pipette, but not for capillary blood transfer [35]. Another report showed similarity between venous and capillary blood enumeration of both CD4 and %CD4 cells using the BD FACSCalibur and the BD FACSCount^™^ systems [22, 39]. Our results for the BD FACSPresto system for CD4, %CD4 T cells, and Hb in venous and capillary blood are similar. The mean %bias and R^2^ values showed equivalency with the predicate methods for the three parameters.

**Fig. (2):**
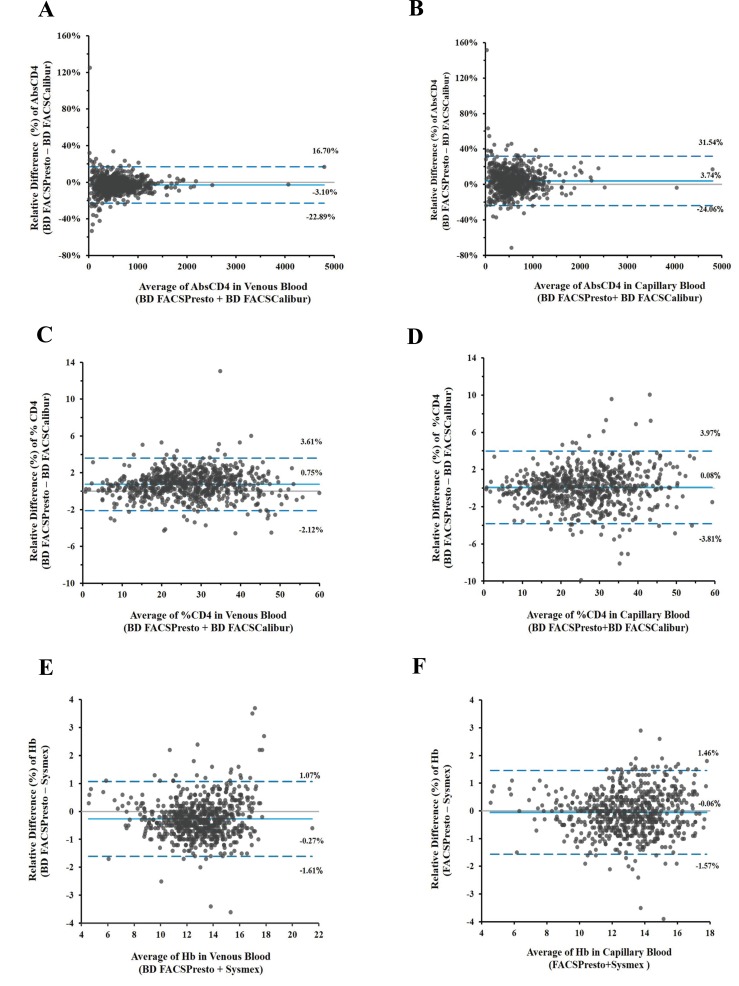
CD4 absolute counts, %CD4, and Hb bias in venous and capillary blood. Bland-Altman plots illustrate the biases for venous (2A, 2C, and 2E) and capillary (2B, 2D, and 2F) samples with limits of agreement. Biases for CD4 cell counts are shown in 2A and 1B, for %CD4 cells in 2C and 2D, and hemoglobin in 2E and 2F. The x-axis displays the average (CD4 counts, %CD4 cells, or Hb) and the y-axis is the difference (CD4 counts, %CD4 cells, or Hb).

**Fig. (3):**
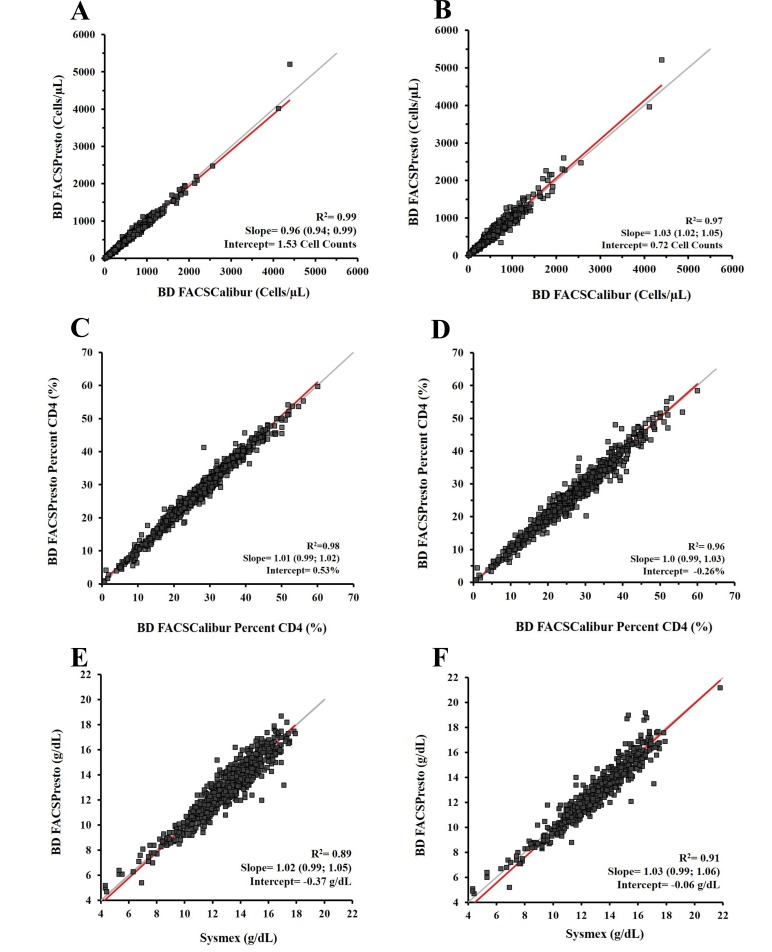
Deming regression analysis, Deming regression plots for CD4 cell counts, %CD4 and Hb in venous and capillary blood. BD FACSPresto vs BD FACSCalibur or Sysmex systems. Deming regression results are depicted for venous blood (3A, 3C, and 3E) and capillary blood (3B, 3D, and 3F). The CD4 count results are shown from weighted Deming regression in 3A and 3B; for %CD4 cells and Hb, the unweighted Deming regression in 3C and 3D and in 3E and 3F respectively. The x-axis displays the predicate method for CD4 cell counts, %CD4 cells, or Hb, and the y-axis corresponds to the BD FACSPresto system. Plots show the R^2^, slope, and the intercept values for each parameter. The identity regression line is in gray and the calculated regression is in red.

A study reported differences in Hb and hematocrit capillary blood measurements [40]. However, the challenge with CD4 counts determination in capillary blood is that the sample must be whole blood to provide valid results. Therefore, the device selected for finger-stick, and the steps to obtain an adequate amount of blood and transfer the blood onto the cartridge, are fundamentally important to attain accurate results. The validated capillary blood collection method used during the study was reproducible, because our results from these two specimen types were equivalent (Figs. **2** and **3**).

The clinically relevant CD4 cell count cutoff of 200 cells/µL was used to analyze the overall, positive, and negative method agreement, similar to results reported at 200 and 350 cells/µL [22]. Venous and capillary sample results from the BD FACSPresto system showed >97% overall agreement, which is relevant for identifying patients eligible for ART in situations in which HIV viral load or the availability of ART drugs is limited. Thus, the healthcare facility may require prioritizing treatment or monitoring subjects with co-infections or opportunistic infections [31, 32]. Similar analyses have been presented as sensitivity and specificity for products in the market [33, 34, 41].

The BD safety engineered, reliable BD Microtainer lancet used during the study has been marketed worldwide. Lancet failures were <1% during the study. In addition, the phlebotomists after short training were effective and successfully transferring the capillary blood directly onto the cartridge. The simplicity and effectiveness of the procedure was reflected in our capillary blood results, supporting the CD4, %CD4 T cells, and Hb equivalency with venous blood.

The BD FACSPresto system performed favorable in terms of throughput and demonstrated low rate of failures (<1.3%). Conservative throughput for the BD FACSPresto system is estimated to be 10 cartridges per hour because the cartridge incubation is staged outside the instrument on the BD FACSPresto workstation and results are available within 25 minutes from blood application, suggesting that the BD FACSPresto system can facilitate a rapid turnaround of the results at the healthcare facilities [22]. The BD FACSPresto cartridge failure rate was <1.3%. A failure was defined as any cartridge or system error that was not be resolved by re-running the cartridge or if the cartridges were not re-run during study testing. Cartridges were re-run only once. Re-running the same cartridge adds ~3-5 min additional minutes to results, so under these conditions the estimated total time is about 30-35 min assuming sequential steps:

Blood draw→Blood transfer to cartridge→Incubation→Results→Rerun

However, when the sample would require re-preparation, the process restarts by dispensing the blood in a new cartridge and incubation. In these cases, preparing a new cartridge might cause delays for clinicians and patients, requiring an additional finger-prick for capillary samples, and increasing cost in extra cartridge(s). Therefore, it is important to ensure that the failure rate is low. The BD FACSPresto system has shown a low failure rate that compares positively with data from similar systems [42].

Our results from the multisite reproducibility and repeatability assessed between laboratory total variability with satisfactory results. Variability between instrument/operator, between cartridge lot, within run and total variability also showed robust results.

The Bland-Altman results reported here provide a solid range of the performance of the system, and a similar method has been used to calculate the total analytical error; however, the outcome of the analysis depends on the CD4 measurement selected. The two different statistical methods, Bland-Altman or total analytical error, can be used to estimate the bias of the system performance in relation to the standard of care, in addition to the Deming Regression used in this report. In this paper, the Bland-Altman statistics were presented as it is widely accepted among clinicians. The study leveraged the routine procedures for specimen collection and sample testing at the study sites. Assessing the impact of biological variability [43] during the study was outside the scope of the study.

There is an ongoing debate about the value of long-term CD4 cell count monitoring in situations when viral load testing is available, and patients are virologically suppressed [44]. Although WHO guidelines for developing countries with high HIV+/AIDS burden recommend only occasional CD4 count testing, typically for baseline and relapse, the CD4 cell count is frequently measured in low-burden countries to provide closer monitoring and better care [31]. The BD FACSPresto system provides a portable capacity for accurate CD4 cell testing in settings where flow cytometry cannot be implemented, which can enable the upgrade of the standard of care in high-burden developing countries by making the close monitoring, including earlier detection of relapse, of high-risk and vulnerable subjects [8, 37, 39, 44, 45] a realistic and practical possibility. CD4 count has been used to prioritize patients in need of treatment and clinical management in places with limited access to ART or lacking viral load testing capacity, or both, and in cases in which the CD4 cells of a few patients might fail to increase despite virological suppression [31, 46].

The BD FACSPresto system offers the flexibility of placing a CD4 count analyzer in remote and/or resource-limited areas closer to patient at risk. This will facilitate to clinicians to provide prompt clinical management by reducing the time to results, which is particularly important for those patients presenting late or returning to care after a period of treatment interruption, and those experiencing virological or clinical failure. Determination of CD4 count continues to be critical in decisions for screening and prophylaxis for major opportunistic infections, and when to stop. Shubber *et al.* [47] have shown that measuring the baseline of CD4 cell count can be used for indication of nevirapine and the potential increased risk of nevirapine-associated hypersensitivity reaction at increased CD4 cells [14]. In summary, the BD FACSPresto system for CD4 testing offers the potential benefit of making accessible the timely CD4 count testing and reducing the time for ART initiation. Additional benefits include services at the primary care level by enabling the clinician to establish the baseline of CD4 cells for patients beginning treatment, provide the correct ART regimen, and reduce patient loss to follow-up. This strategy can have a positive impact in public health and offer excellent value for immunologic staging across a range of parameters in resource-limited settings. Recently, a meta-analysis of the POC CD4 testing has shown an increased on retention from HIV/CD4 testing, reducing result turn-round time, ART timely initiation, cost-effective and acceptable [48].

Data from this study should be interpreted in light of the following limitations: the study was carried out in central laboratory facilities, and the BD FACSPresto instruments were located at facilities broadly representative of central laboratory settings. In these laboratory settings, the CD4 cell count testing done by laboratory staff and laboratory facilities requires strong capacity–building among staff and constant supervision to ensure high quality sample collection and testing. Our results obtained from the BD FACSPresto system and BD FACSCalibur with BD Tritest CD3/CD4/CD45 reagent and Sysmex system are equivalent. Although the results of this study might not be generalizable to different laboratory settings or health facilities in resource-limited settings.

## CONCLUSION

In conclusion, the BD FACSPresto system is a robust and reliable system that provides accurate results for capillary or venous samples for measuring CD4 and %CD4 T cells in the lymphocyte population. The BD FACSPresto is an easy-to-use alternative requiring minimal training for optimally generating CD4 cells and Hb integrated results in healthcare facilities in resource-limited settings. This multisite evaluation demonstrated equivalency of performance between the BD FACSPresto, the BD FACSCalibur and the Sysmex systems for calculation of CD4, %CD4 T cells, and Hb in venous and capillary blood. The BD FACSPresto system also displayed robust precision results across three sites during testing. This product was CE Marked (IVD Directive 98/79/EC) and WHO prequalified in 2014, and received FDA clearance in 2015.

## Figures and Tables

**Table 1:** Method comparison enrolled cohort summary by specimen type, parameter and age group.

**Specimen Type**	**Parameter**	**Age Group**	**N**	**Age Mean**	**Parameter** **Median**	**Parameter** **(Min-Max)**
**Venous**	**CD4 (cells/µL)**	Children	56	7.1	1181	329-4020
		Adolescence	68	15.6	725	5-5204
		Adult	592	42.7	491	14-1624
	**CD4%**	Children	56	7.1	34.28	11.15-53.77
		Adolescence	68	15.6	29.775	0.63-50.02
		Adult	592	42.7	25.875	0.96-59.82
	**Hb (g/dL)**	Children	56	7.1	11.4	8.6-14.9
		Adolescence	68	15.6	12.1	6.8-16.7
		Adult	596	42.7	13.2	4.7-21.2
**Capillary**	**CD4 (cells/µL)**	Children	53	7.0	1157	306-3969
		Adolescence	65	15.6	748	8-5216
		Adult	563	42.6	510	8-1509
	**CD4%**	Children	53	7.0	32.16	11.86-56.2
		Adolescence	65	15.6	28.65	0.73-47.34
		Adult	563	42.6	25.12	0.57-58.5
	**Hb (g/dL)**	Children	53	7.0	11.8	8.8-14.5
		Adolescence	66	15.6	12.8	7.1-17
		Adult	572	42.6	13.3	4.7-18.7

**Table 2:** Bland-Altman and Deming regression summary results in venous and capillary blood.

**Specimen Type**	**Parameter**	**N**	**Bland-Altman**		**Deming Regression**			
			**Mean %Bias**	**95% LOA** **(LL, UL)**	**R^2^**	**Slope (95% CL)**		**Intercept**
**Venous Blood**	**CD4 (cells/µL)**	716	3.10	(-22.89, 16.70)	0.99	0.96	(0.94–0.99)	1.53 counts
	**%CD4**	716	0.75	(-2.12, 3.61)	0.98	1.01	(0.99–1.02)	0.0%
	**Hb (g/dL)**	720	-0.27	(-1.61, 1.07)	0.91	1.03	(0.99–1.06)	-0.06 g/dL
**Capillary Blood**	**CD4** **(cells/µL)**	681	3.74	(-24.06, 31.54)	0.97	1.03	(1.02–1.05)	0.72 counts
	**%CD4**	681	0.08	(-3.81, 3.97)	0.96	1.00	(0.99–1.03)	-0.26%
	**Hb (g/dL)**	691	-0.06	(-1.57, 1.46)	0.89	1.02	(0.99–1.05)	-0.37 g/dL

LOA= limits of agreement; LL= lower limit; UL= upper limit

**Table 3:** Paired venous and capillary summary.

**Parameter**	**N**	**Results**
**CD4** **(cells/µL)**	678	Slope: 1.07 (1.05, 1.09)
		Intercept: 0.58 counts
**%CD4**	678	Slope: 1.00 (0.99, 1.02)
		Intercept: -0.80%
**Hb (g/dL)**	685	Slope: 1.00 (0.97, 1.02)
		Intercept: 0.23 g/dL

**Table 4:** Agreement at CD4 of 200 cells/µL in venous and capillary blood.

**Method**	**Venous blood**			Capillary blood		
Test (BD FACSPresto)	**Positive (<200)**	**Negative (>200)**	**Total**	**Positive (<200)**	**Negative (>200)**	**Total**
**Positive (<200)**	75	10	85	62	5	67
**Negative (<200)**	1	631	632	11	604	615
**Total**	76	641	717	73	609	682
**Percent (%) Agreement**			**95% CL**		**LL, UL**	
**Venous Blood**		Overall	98.50%		97.27, 99.14	
		Positive	98.70%		92.89, 99.97	
		Negative	98.40%		97.48, 99.40	
**Capillary Blood**		Overall	97.70%		96.22, 99.55	
		Positive	84.90%		76.73, 93.14	
		Negative	99.20%		98.46, 99.90	

LL= Lower limit; UL= Upper Limit

**Table 5:** Estimated total precision for between–laboratory variability.

**Process Control**	**BCW**		**MED**		**SFG**	
	**Mean**	**%CV(UL)**	**Mean**	**%CV(UL)**	**Mean**	**%CV(UL)**
**CD4 Low**	137.97	5.23 (6.39)	138.77	5.79 (7.06)	140.07	5.12 (6.24)
**CD4 Normal**	844.67	2.87 (3.50)	844	2.49 (3.04)	856.30	1.88 (2.29)
**-**	**Mean**	**SD(UL)/** **%CV(UL)**	**Mean**	**SD(UL)/** **%CV(UL)**	**Mean**	**SD(UL)/** **%CV(UL)**
**%CD4 Low**	12.65	0.62 (0.79)	12.85	0.59 (0.76)	12.76	0.56 (0.72)
**%CD4 Normal**	43.96	1.82 (2.22)	44.08	1.79 (2.19)	44.42	1.59 (1.93)
**-**	**Mean**	**%CV(UL)**	**Mean**	**%CV(UL)**	**Mean**	**%CV(UL)**
**Hb Level 1**	7.20	3.11 (3.79)	7.26	2.18 (2.66)	7.23	2.83 (3.46)
**Hb Level 2**	12.93	1.52 (1.86)	12.94	1.32 (1.61)	12.94	1.71 (2.08)
**Hb Level 3**	17.11	1.34 (1.64)	17.06	1.30 (1.58)	17.20	1.02 (1.24)

%CV= Percent coefficient of variation; UL= Upper limit; BCW= BloodCenter of Wisconsin; MED= BD Biosciences MedLab; SFG= San Francisco General Hospital.

**Table 6:** Patient sample repeatability.

**CD4 Bin (cells/ µL) / Parameter**	**Mean**	**Between Instrument / Operator**		**Between Lot**		**Within Run**		**Total**	
		**SD**	**%CV**	**SD**	**%CV**	**SD**	**%CV**	**%CV**	**%CV UL**
**CD4 ≤200**	97.26	2.43	2.49	0.79	0.81	6.94	7.13	7.60	8.35
**CD4 >200 to ≤500**	327.02	4.45	1.36	4.76	1.46	17.28	5.28	5.65	6.11
**CD4 >500 to ≤1,000**	691.35	5.53	0.8	0.57	0.08	22.25	3.22	3.32	3.54
**>1,000 to <5,000**	1367	10.95	0.8	7.9	0.58	32.39	2.37	2.57	2.81
**CD4 (cells/ µL)**	623.6	6.38	1.02	4.25	0.68	21.74	3.49	3.27	3.85
**%CD4**	26.92	0.19	0.69	0.1	0.37	0.72	2.69	2.8	2.91
**Hb (g/ dL)**	13.49	0.55	4.11	0.11	0.85	0.39	2.92	5.11	5.32

%CV= Percent coefficient of variation; UL= Upper limit
